# Mental Health and Functioning of Female Sex Workers in Chittagong, Bangladesh

**DOI:** 10.3389/fpsyt.2015.00176

**Published:** 2015-12-15

**Authors:** Michael P. Hengartner, Md Nazrul Islam, Helene Haker, Wulf Rössler

**Affiliations:** ^1^Department of Applied Psychology, Zurich University of Applied Sciences, Zurich, Switzerland; ^2^Rehabilitation Centre for Prostitutes and Rootless Children (PARC), Chittagong, Bangladesh; ^3^Translational Neuromodeling Unit, Institute for Biomedical Engineering, University of Zurich and ETH Zurich, Zurich, Switzerland; ^4^Department of Psychiatry, Psychotherapy and Psychosomatics, University of Zurich, Zurich, Switzerland; ^5^Laboratory of Neuroscience (LIM 27), Institute of Psychiatry, University of São Paulo, São Paulo, Brazil

**Keywords:** sex work, prostitution, mental health, psychopathology, epidemiology, community psychiatry

## Abstract

**Aim:**

To examine the mental health and functioning of female sex workers (FSW) in Chittagong, Bangladesh, a population that has commonly been neglected in mental health policy and research.

**Methods:**

We included 259 women in the study (*M* age: 23.2 years; range: 11–48). The comprehensive Composite International Diagnostic Interview was used to assess their 12-month prevalence rates of DSM-IV mental disorders, and a comprehensive questionnaire was adapted to explore various factors, such as socio-demographics, working and living conditions, or experiences of abuse.

**Results:**

On average, participants began their commercial sex work at 18.5 years old (range: 10–45). Their main motives for sex work were coercion (49.4%) and the necessity to financially support families (54.8%). In total, 224 FSW (86.5%) wanted to quit commercial sex work. A mental disorder within the past 12 months was reported by 100 FSW (38.6%), with drug abuse clearly being the most prevalent diagnosis (15.4%). Sexual, physical, and emotional abuse were very common among the FSW, and substance use disorders (SUD) were significantly more prevalent in persons who experienced emotional abuse (OR = 2.2). Prevalence rates of any mental disorder and SUD were higher in women who did sex work to support their family, whereas mood disorders were more frequent in those who needed the money to pay debts. Participants with any disorder were significantly older than those without (*M* age: 24.4 vs. 22.5 years) and had started significantly later in the sex business (*M* age: 19.7 vs. 17.7 years).

**Conclusion:**

Our study revealed that FSW in Chittagong are very vulnerable and highly impaired, as expressed by high rates of abuse and mental disorders. Coercion is very common and many FSW are required to work in the sex business because they need the money to support their families. FSW are a very marginalized population, especially in the developing countries where awareness for mental health is low and the availability of mental health services is insufficient.

## Introduction

As a disadvantaged and marginalized population, female sex workers (FSW) frequently suffer from coercion, stigmatization, and severe health impairments (1, 2). From a public health perspective, most research has focused on sexually transmitted diseases and drug abuse, but less so on mental health and contributing psychological factors. To estimate the burden of sex work on global public health, comprehensive investigations of this vulnerable and often victimized population are urgently needed (3). Moreover, because sex work is highly dependent on socio-cultural and economic contexts, evaluations are required that focus on populations other than in Western countries.

In a study of FSW in Eastern Nepal (*n* = 210), the prevalence of depression was 82.4% (4). The occurrence of physical abuse was strongly related to increased rates of depression, but no associated effect was found for rape or sexual abuse. However, in this study, the diagnosis of depression was based on a simple cut-off value derived from a screening instrument. In a small study (*n* = 55) of FSW in Israel (5), 17% of women showed signs of post-traumatic stress disorder and 19% screened positive for major depression. However, again only screening questionnaires were applied to assess mental health. Using an age-matched control design with 29 FSW from Dunedin and Wellington, New Zealand, Romans et al. (6) found that the frequency of mental health problems was not higher for their FSW sample than for the general population, whereas exposure to adult physical and sexual abuse was increased for the former group. The validity of this study was limited by the very small number of FSW participants and by the reliance upon a self-report screening questionnaire to assess mental health problems.

Only a few quantitative studies have utilized reasonably large samples and, most importantly, structured clinical interviews, for systematically assessing mental health among FSW. One of these is work by Rössler and colleagues (7), which revealed a 12-month prevalence rate for any disorder of 50.3%, with major depression (24.4%) and specific phobia (17.6%) being the most prevalent single disorders in 193 FSW from Zurich, Switzerland. Positive associations with any type of mental disorder included violence and rape in the sex business, the number of working days per week, or a lack of social support.

The present study was designed to adapt the methodology used in that Zurich evaluation and to apply that approach to a sample within a country that differs both socio-economically and culturally from Switzerland. Our main objective is to cross-validate the relationship between sex work and mental health problems in a sample of FSW from Bangladesh. Although our investigation was mostly exploratory, we hypothesized that the prevalence rates of mental disorders would be considerably higher for FSW than for the general population, based on outcomes previously reported in the scientific literature.

## Materials and Methods

### Participants and Procedure

The data sampling was conducted by the Rehabilitation Centre for Prostitutes and Rootless Children (PARC) in Chittagong, Bangladesh. This Non-Governmental Organization (NGO) is dedicated to improve the situation of sex workers and homeless children and youths by providing support, education, and access to health services. The total number of FSW in Chittagong is unknown but is estimated to be at least 6000. With the help of some FSW who collaborate closely with PARC, a local study coordinator identified approximately 600 FSW from the various localities and facilities known to be frequently attended by FSW, of whom 350 were randomly contacted directly at these different locations within Chittagong. Of the 350 women who were approached and introduced to the study aims, 302 agreed to participate (response rate: 86.3%). Eventually, 43 dropped out because they did not complete either the psychiatric assessment or the basic questionnaire, resulting in a final sample size of *n* = 259 (response rate: 74.0%). Interviews were conducted between September 2011 and February 2012 by five specially trained research associates at PARC offices, and all participants gave their fully informed consent (depending on their level of literacy written or verbal). Depending on the duration of the interview, all participants received a small expense allowance of 250 (if completed in 1 day) to 350 (if completed in up to 3 days) Bangladeshi Taka after completion of the interviews (corresponds to approximately 2.5–3.5 US$). This study was ethically approved by the responsible Bureau of NGO Affairs of the Government of the Peoples’ Republic of Bangladesh (reference number No-Abbue/A-4/PARC/151-3/2011-1547) on 21 June 2011 and was conducted in accordance with the declaration of Helsinki of the World Medical Association.

### Instruments and Measures

The basic questionnaire was adapted from one previously used for a study of FSW in Zurich, Switzerland (7). This fully structured questionnaire gathered socio-demographic data, including information about working conditions and motivation to participate in sex work. The questionnaire was carefully evaluated by the local investigator of the study group (Md Nazrul Islam) before application to the study participants to ensure that the translated version conformed to Bangladeshi socio-cultural characteristics. All questions were answered according to predefined response categories (mostly “yes” or “no”). The occurrence of sexual abuse was assessed by asking the respondents “were you ever raped or sexually abused?” Physical abuse was addressed by asking “have you ever been hit, beaten, or physically injured?,” while emotional abuse was evaluated with the query “have you ever been set under pressure, controlled, or threatened?.” All three forms of abuse were rated on a four-point Likert scale ranging from “never,” “seldom,” “often,” to “very often.”

Psychiatric diagnoses were provided by the fully structured World Health Organization (WHO) Mental Health Composite International Diagnostic Interview (MH-CIDI) (8). The MH-CIDI determines the presence of mental disorders within the past 12 months according to the definitions and criteria of the DSM-IV (9) and has demonstrated acceptable reliability and validity (10, 11). Those diagnoses considered here are comprised of mood disorders (major depression disorder, dysthymia, recurrent brief depression, or bipolar disorders I and II), anxiety disorders (agoraphobia, panic disorder, generalized anxiety disorder, obsessive–compulsive disorder, post-traumatic stress disorder, social phobia, or specific phobia), substance use disorders (SUD) (alcohol abuse and dependence or drug abuse and dependence), and impulse-control disorders (conduct disorder, intermittent explosive disorder, attention deficit disorder, oppositional defiant disorder, anorexia, or bulimia nervosa). The second author of the present study (Md Nazrul Islam) was extensively trained in the application of the MH-CIDI at the University of Michigan (Ann Arbor, MI, USA). The original English version of the MH-CIDI was translated in Bengali by a Bangladeshi English Professor. Afterwards, the translation was edited, culturally adjusted, and field tested by mental health professionals, including Md Nazrul Islam, for use in Bangladesh. The Bengali translation of the MH-CIDI was officially approved by the University of Michigan and the authors. In addition to provide a translation of the English version for use in Bangladesh, Md Nazrul Islam trained 15 lay interviewers in Chittagong, from whom the five best suited were selected according to their level of performance, accuracy, and patience for conducting the actual interviews.

### Statistical Analysis

Associations between multinomial variables were examined with contingency tables and χ^2^ tests. The ordered categorical variables for abuse were entered as dependent variables and the diagnostic variables as predictors into a series of categorical regression analyses. Those associations were additionally cross-validated by applying contingency tables and χ^2^ tests. Associations with continuous variables, such as age or income, were examined with a series of one-way ANOVAs. All analyses were performed with SPSS 21 for Windows.

## Results

The basic characteristics of the FSW are shown in Table 1. Most of the participants were uneducated, single, and had no other job besides prostitution. The mean age at which these persons had begun their sex work was 18.5 years, with some women starting as early as 10 years old. Mean income varied greatly, with the SD being almost as high as the mean. Only a minority worked in a sex club or brothel (8.1%); most served in their clients’ residences (61.8%) or in residential hotels (63.7%), indicating that the majority acquired their customers on the streets. In all, 224 women (86.5%) expressed a desire to quit sex work. By far, the most frequent responses to the question of why they worked as FSW were “to support my family” (*n* = 142; 54.8%) and “because I was forced into it” (*n* = 128; 49.4%). Only 15 women (5.8%) answered, “Because I like it.” Several response options were possible. Social support was generally low, with only 58 women (22.4%) indicating that they had a trusted person upon whom they could rely and 52 (20.1%) saying that they knew someone who was there to help when needed. A total of 43 women (16.6%) sometimes used drugs in order to perform their work, while another 37 (14.3%) stated that their drug use was often or very often.

**Table 1:** **Characteristics of study participants**.

Categorical variables	Category	*N* (%)
Education	No	199 (77.0)
	Yes	59 (23.0)
Committed relationship	No	199 (76.8)
	Yes	60 (23.2)
Children	No	104 (40.2)
	Yes	155 (59.8)
Other job besides sex work	No	205 (79.2)
	Yes	54 (20.8)
Continuous variables	Range	Mean (SD)
Age	11.0–47.8	23.2 (6.7)
Age at onset of sex work	10.0–45.0	18.5 (4.9)
Workdays per week	2–7	4.5 (1.4)
Clients per week	2–80	9.6 (8.0)
Monthly income from sex work (in BDT)	0.0–60,000.0	9665.6 (8605.5)

*BDT, Bangladeshi Taka; 1 BDT ≈ 0.01 US$ (as of 2011)*.

The 12-month prevalence rates of common mental disorders, according to DSM-IV criteria, are shown in Table 2. Mood disorders were reported for 11 participants (4.2%), with all of them meeting the diagnosis of major depression disorder. Markedly more prevalent was anxiety disorders (*n* = 54; 20.8%), with the most frequent diagnoses being agoraphobia (*n* = 18; 6.9%) and obsessive–compulsive disorder (*n* = 27; 10.4%). The most prevalent cluster overall was that of SUD (*n* = 57; 22.0%). Here, drug abuse was the most frequently met single diagnosis (*n* = 40; 15.4%). Finally, impulse-control disorders were determined for 24 participants (9.3%), with intermittent explosive disorder being the most commonly diagnosed (*n* = 19; 7.3%). A mental disorder from within any group described above was reported by 100 participants (38.6%). The number of co-occurring disorders was 1 for 46 persons (17.8%), 2 for 29 (11.2%), and 3 or more for 25 (9.7%).

**Table 2:** **12-month Prevalence rates of DSM-IV mental disorders**.

Mood disorder		Anxiety disorder		Substance use disorder		Impulse-control disorder	
Diagnosis	*N* (%)	Diagnosis	*N* (%)	Diagnosis	*N* (%)	Diagnosis	*N* (%)
MDD	11 (4.2)	Agoraphobia	18 (6.9)	Alcohol abuse	21 (8.1)	CD	0 (0.0)
Dysthymia	0 (0.0)	Panic disorder	1 (0.4)	Alcohol dependence	16 (6.2)	IED	19 (7.3)
RCB	0 (0.0)	GAD	0 (0.0)	Drug abuse	40 (15.4)	ADD	4 (1.5)
BP I	0 (0.0)	OCD	27 (10.4)	Drug dependence	25 (9.7)	ODD	2 (0.8)
BP II	0 (0.0)	PTSD	8 (3.1)			Anorexia nervosa	0 (0.0)
		Social phobia	9 (3.5)			Bulimia nervosa	0 (0.0)
		Specific phobia	0 (0.0)				
Any mood disorder	11 (4.2)	Any anxiety disorder	54 (20.8)	Any SUD	57 (22.0)	Any ICD	24 (9.3)

*MDD, major depression disorder; RCB, recurrent brief depression; BP I/II, bipolar disorder I/II; GAD, generalized anxiety disorder; OCD, obsessive–compulsive disorder; PTSD, post-traumatic stress disorder; CD, conduct disorder; IED, intermittent explosive disorder; ADD, attention deficit disorder; ODD, oppositional defiant disorder*.

**Figure 1:**
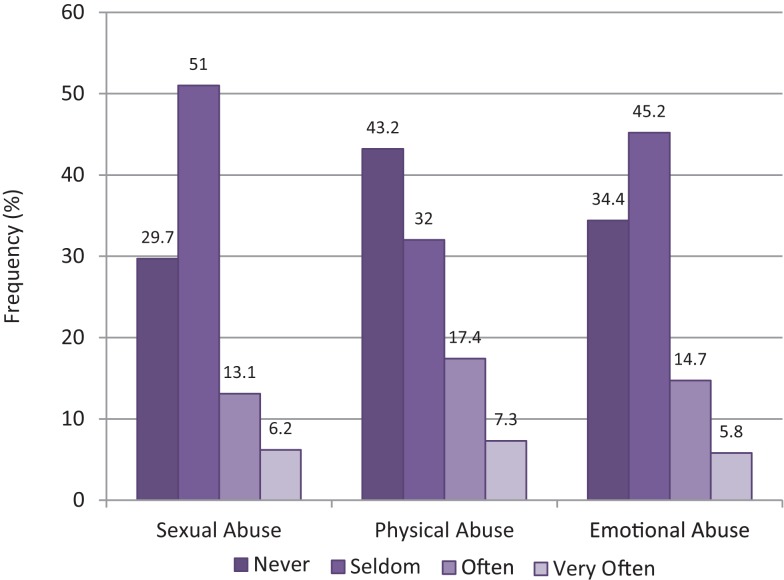
**Self-reported abuse**.

Exposure to some form of abuse was common among FSW (Figure 1), with approximately 20% each saying that they were often or very often sexually abused (*n* = 50; 19.3%), physically abused (*n* = 64; 24.7%), or emotionally abused (*n* = 53; 20.5%). Contrary to our expectations, however, no diagnosis of mood, anxiety, SUD, or impulse-control disorder was significantly related to the occurrence of sexual or physical abuse. However, SUD related to emotional abuse, indicating that women who experienced threats and coercion were more likely to have SUD (OR = 2.2; 95% CI = 1.3–3.8; *p* = 0.005).

The prevalence of mental disorders related to different motives to work in the commercial sex business is shown in Table 3. The presence of any type of disorder was *less* common in women who were forced into the business but more prevalent in those women who did this work to support their families. Mood disorders were more frequent in women who did the job to pay their debts, whereas SUD were more common in those FSW who were required to support their families.

**Table 3:** **Relationship between prevalence of DSM-IV mental disorders and reported motives for employment as an FSW**.

	Any disorder	Mood disorder	Anxiety disorder	Substance use disorder	Impulse-control disorder
To finance my education	No: 38.5%	No: 4.1%	No: 20.9%	No: 23.0%	No: 9.0%
	Yes: 40.0%	Yes: 6.7%	Yes: 20.0%	Yes: 6.7%	Yes: 13.3%
Because I could not find any other job	No: 39.2%	No: 3.8%	No: 22.0%	No: 22.0%	No: 9.7%
	Yes: 37.0%	Yes: 5.5%	Yes: 17.8%	Yes: 21.9%	Yes: 8.2%
Because I was forced into it	**No: 45.8%**	No: 3.8%	No: 24.4%	No: 25.2%	No: 10.7%
	**Yes: 31.2%**	Yes: 4.7%	Yes: 17.2%	Yes: 18.8%	Yes: 7.8%
To support my family	**No: 29.9%**	No: 3.4%	No: 18.8%	**No: 16.2%**	No: 7.7%
	**Yes: 45.8%**	Yes: 4.9%	Yes: 22.5%	**Yes: 26.8%**	Yes: 10.6%
To pay debts	No: 37.4%	**No: 2.7%**	No: 19.4%	No: 21.6%	No: 8.6%
	Yes: 45.9%	**Yes: 13.5%**	Yes: 29.7%	Yes: 24.3%	Yes: 13.5%
To finance drugs	No: 37.6%	No: 3.9%	No: 20.1%	No: 21.4%	No: 8.3%
	Yes: 46.7%	Yes: 6.7%	Yes: 26.7%	Yes: 26.7%	Yes: 16.7%
To support my partner	No: 38.6%	No: 4.4%	No: 20.3%	No: 21.9%	No: 9.2%
	Yes: 37.5%	Yes: 0.0%	Yes: 37.5%	Yes: 25.0%	Yes: 12.5%
Because I like it	No: 38.5%	No: 4.5%	No: 21.3%	No: 20.9%	No: 9.8%
	Yes: 40.0%	Yes: 0.0%	Yes: 13.3%	Yes: 40.0%	Yes: 0.0%

*Bold significance level is *p* < 0.05*.

**Figure 2:**
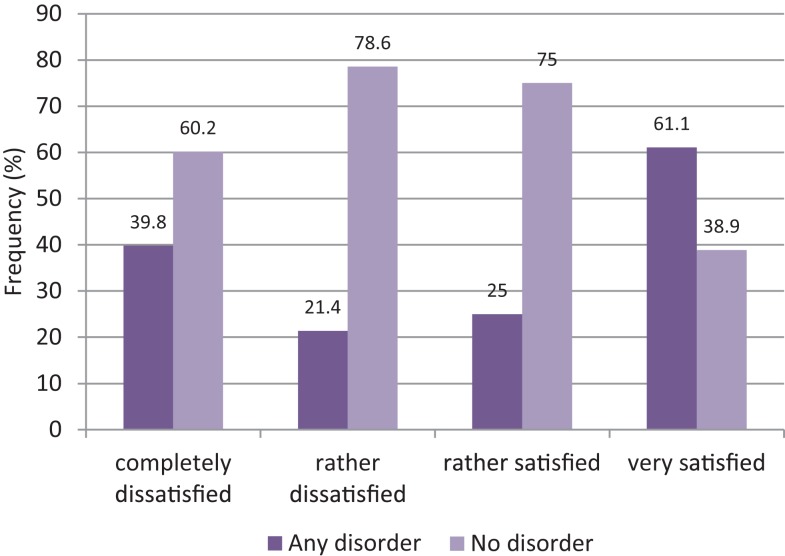
**Relationship between quality of life and occurrence of any mental disorder**.

The feelings of being rejected or avoided by others were not related to either any particular mental disorder or one of the groups of disorders. On a descriptive level, the prevalence of any mental disorder revealed a clear U-shaped distribution across categories of quality of life, with increased prevalence rates at both the negative (completely dissatisfied) and, even more pronounced, the positive (very satisfied) poles (Figure 2). Except for mood disorders, the same trend was found in all disorder groups. Mood disorder occurred only in persons at the lower end of the scale, i.e., accounting for 5.0% in women who were completely dissatisfied and 3.6% in those who were rather unsatisfied.

**Figure 3:**
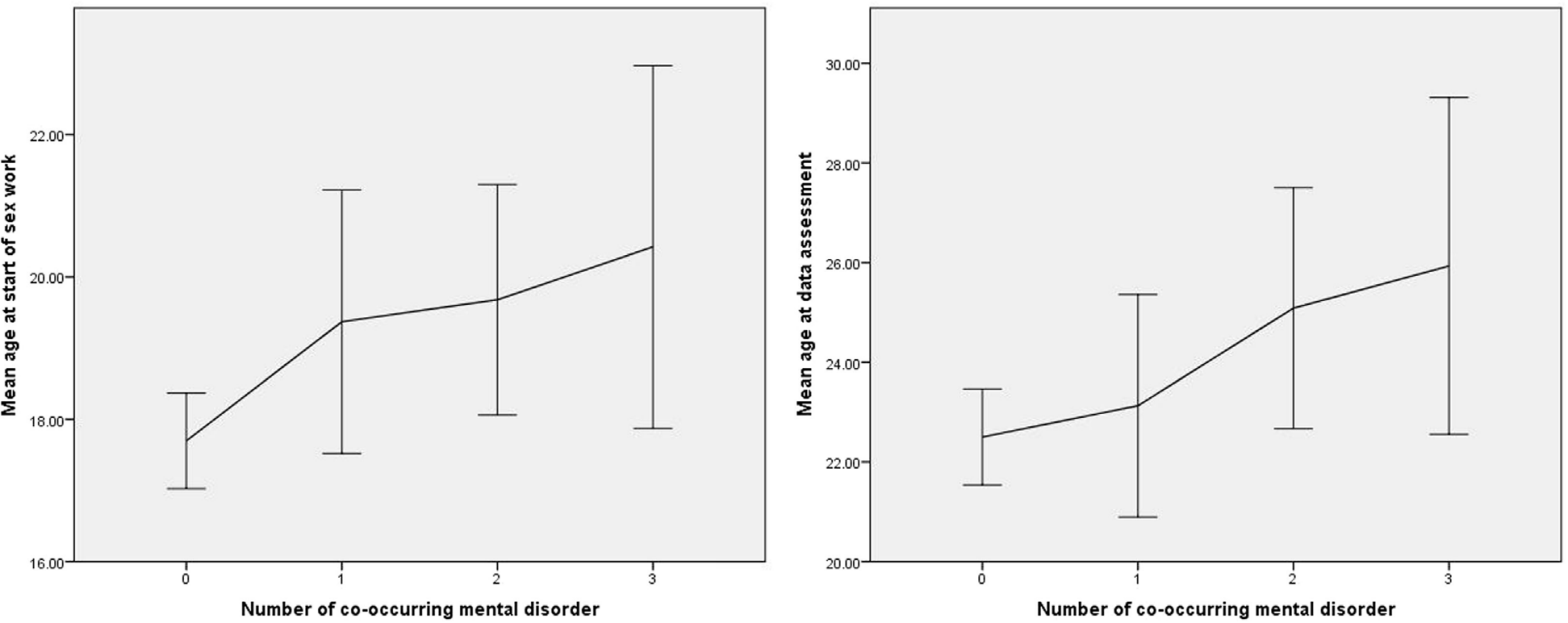
**Association of age with number of co-occurring mental disorders (error bars indicate 95% confidence intervals)**.

Level of education, relationship status, having children, or details of sex work, i.e., monthly income, numbers of clients or days worked per week, were not related to any disorder group. However, the current age of a participant and the age at which she began sex work were significantly associated with the prevalence of any mental disorder. Those who met criteria for any type of disorder were significantly older (*M* age: 24.4 vs. 22.5; *F* = 4.97, df = 1, *p* = 0.027) and had started at a significantly later age in the business (*M* age: 19.7 vs. 17.7; *F* = 10.59, df = 1, *p* = 0.001). Moreover, a polynomial contrast analysis indicated that both current age (*F* = 7.18, df = 1, *p* = 0.008) and the age at which sex work began (*F* = 6.39, df = 1, *p* = 0.012) showed a linear increase with respect to the number of co-occurring disorders (Figure 3).

## Discussion

The aim of this study is to provide a comprehensive survey of mental health problems and contributing psychological factors within a rather large sample of FSW in Chittagong, Bangladesh. Although some individuals voluntarily enter the commercial sex industry, most are forced. Accordingly, these women display a wide variety of characteristics and motivations. A small minority of autonomous women are satisfied with their jobs, experience little burden, and enjoy potentially high incomes. As shown by Spice (2) and Rössler et al. (7), FSW are a very heterogeneous population with varying backgrounds and life histories that range from those employed in expensive studios, who enjoy this work and have few burdens, to very miserable and exploited women with very high burdens who acquire their clients on the streets. Persons in the sample described here tend to belong to the latter type. In contrast to a high-functioning minority of autonomous FSW, a substantial proportion are illegally trafficked, drug addicted, and/or driven into sex work by poverty, coercion, or abuse. Those women typically have high rates of mental disorders, are in poor general health, and experience abundant violence, coercion, and exploitation (4, 7, 12–14).

Overall, the present FSW sample from Chittagong was very vulnerable and impaired, and most women participated only reluctantly in the commercial sex business. The vast majority (86.5%) indicated that they wanted to quit sex work, 49.4% were forced into the industry, and only 5.8% reported that they were involved in commercial sex work because they liked it. The 12-month prevalence rate for any disorder was 38.6%, meaning that more than one out of three women suffered from severe psychopathology. However, and most importantly, even this alarmingly high prevalence rate necessarily underestimates the true impact of mental health problems, since various severe mental disorders, such as personality disorders and psychotic disorders, were not assessed. A recent review on mental disorders in Bangladesh estimated that the rate of mental disorders based on structured interviews and community samples varies between 12.2 and 16.1% in the general population (15), which lies well within the interquartile range for 12-month prevalence for mental disorders worldwide (16). Karim and colleagues (17) have reported a prevalence rate of 12.2% for any mental disorder across three districts from Dhaka in Bangladesh. Our 12-month estimate of 38.6% for any mental disorder in this sample of FSW is well above the prevalence rate measured in the general population, thereby supporting our hypothesis. Furthermore, the high prevalence of mental disorders in FSW has been confirmed by other studies that used sufficiently large samples from distinct countries, such as Switzerland (7) and Nepal (4).

In particular, SUD were considerably common in the present study (12-month prevalence of 22%), which is in accordance with previous studies (12, 18, 19). If one considers that SUD among FSW increases their risk for various afflictions, including sexually transmitted diseases, exploitation, and violence (1, 14), then the extensive use of alcohol and drugs poses a particular threat to their physical health and well-being. Although earlier research has shown that sex work can be a particular means by which one can finance a substance dependency (13, 19, 20), we did not find an association between SUD and the self-reported need to obtain money for drug purchases. Thus, although SUD are very frequent in FSW, they are not the main reason for why one enters the sex business. This finding is congruent with a thorough review by Vanwesenbeeck (21), who stressed that substance use appears to be a causal risk factor for sex work only in specific subgroups of street-working FSW, such as homeless youths, but not in other FSW subgroups.

Abuse and maltreatment are very common in various FSW subgroups, although not in all (6, 7, 12). In the present study, 19.3, 24.7, and 20.5% of all participants reported that they often or very often experienced severe sexual, physical, or emotional abuse. Those numbers illustrate the marked vulnerability of many FSW and the need for both prevention and intervention campaigns for those women. We also found a particular association between emotional abuse and SUD, which is in accord with the notion that substance use places certain FSW at greater risk for abuse and coercion (2, 21). Age was positively related to the prevalence and co-occurrence of mental disorders, indicating that younger FSW are more resilient than older ones, or, alternatively, that older women show heightened reactivity to stress. One explanation might be that the pernicious effects of the sex business accumulate over time until the psychological resources of a sex worker are exhausted and she is no longer able to cope with such adversities. This is similar to the findings of Mroczek and Almeida (22). This possibility also implies that the risk for a mental disorder increases with the period of time that someone has been part of this industry.

Another striking finding is the *lower* prevalence of any mental disorder in women who were forced to work in this business when compared to women who worked voluntarily as FSW. That is, FSW who are compelled to enter the sex industry because of other factors than coercion were more likely to develop a mental disorder, or, alternatively, were driven into the sex business because of a pre-existing mental disorder. Increased psychopathology in FSW who were *not* coerced might be explained by either control theory (23) or cognitive dissonance (24). Both theories posit that a conflict between goals/ideals (i.e., “sex work is reprehensible”) and actual behavior (i.e., “I am voluntarily a female sex worker”) may elicit bothersome or aversive affects, hence mental disorders. Two motives related to increased rates of mental disorders revealed here were the need to support one’s family and the pressure to pay debts. Presumably, psychological strain is more impairing and burdensome than pure physical coercion, because psychological resources form the fundament for mental well-being. Therefore, as long as those foundations are not compromised, many psychological mechanisms can be implemented to foster mental resistance and enhance resilience, as discussed previously (25, 26). Thus, not only are interventions required that reduce harm to FSW (1) but more research is also needed that is specifically aimed at the roles of resilience and protective factors in the face of adversity.

Finally, we were surprised to find that, among FSW who were completely dissatisfied with their quality of life, the majority (60.2%) had no mental disorder, whereas in the subgroup of FSW who were very satisfied with their life, the majority (61.1%) met the criteria for any mental disorder. We do not know whether this finding is due to a distorted perception and a reduced self-awareness in women with severe mental disorders, or whether it truly reflects a positive relationship between satisfaction and psychopathology, which would appear counterintuitive. In the literature, there is compelling evidence for a linear negative association between psychopathology and quality of life, that is, the less someone is impaired, the higher the quality of life (27), but the literature also reveals that there is little agreement between patients’ self-reports and objective measures as well as clinician-rated quality of life. Patients’ subjective perception of their quality of life is often distorted by various fallacies and altered affective or cognitive states (28). For instance, many persons with SUD or manic patients markedly overestimate their subjective quality of life. We therefore suggest that reports of high quality of life in persons with severe psychopathology are mainly due to distorted perception and altered self-awareness.

## Conclusion

The two major limitations of this study were its cross-sectional design, which precludes causal reasoning, and our reliance on self-report instruments, which may have biased the results through method effects, such as reduced self-awareness or social desirability. Nevertheless, despite these shortcomings, the present study provides further evidence that, in less developed countries, FSW comprise mostly street workers who are among the most impaired and vulnerable populations worldwide (1, 3). In developing countries, the reliance upon mental health services for severe illness is substantially lower than in Western countries (16, 29). With respect to Bangladesh, Hossain and colleagues (15) have specifically noted that service delivery and provision of adequate treatments for mental disorders is largely insufficient. Prevention programs and harm-reduction campaigns are urgently needed for FSW who are regularly exploited, stigmatized, and marginalized, and who are even more vulnerable because of their poor physical and mental health (1). Although FSW require particular attention, they are readily criminalized and ignored in public and occupational health policies (2). Therefore, as experts and opinion leaders in the field of public (mental) health, we must recognize that FSW urgently need professional help and access to psychiatric treatments, social support, and health services.

## Author Contributions

MH conducted all of the statistical analysis and wrote the manuscript. MI contributed significantly to the conduct of the study and data collection, and critically revised the manuscript. HH and WR conceived and designed the study, instructed MI and his local team about conducting the study, and critically revised the manuscript. All authors have read and accepted the final version of the manuscript.

## Conflict of Interest Statement

The authors declare that the research was conducted in the absence of any commercial or financial relationships that could be construed as a potential conflict of interest.
